# Structural modulation of the gut microbiota and the relationship with body weight: compared evaluation of liraglutide and saxagliptin treatment

**DOI:** 10.1038/srep33251

**Published:** 2016-09-16

**Authors:** Lin Wang, Peicheng Li, Zhaosheng Tang, Xinfeng Yan, Bo Feng

**Affiliations:** 1 Department of Metabolism and Endocrinology, Shanghai East Hospital, Tongji University School of Medicine, Shanghai 200120, People’s Republic of China

## Abstract

The mechanisms underlying the weight-loss effect of GLP-1 receptor agonists need further elucidation. The present study was performed to explore the effects of liraglutide and saxagliptin on the composition of the gut microbiota. Mice were randomly treated with saxagliptin or liraglutide for eight weeks. Their metabolic profiles were assessed, and 454 pyrosequencing of 16s rRNA of faeces was performed. Liraglutide induced a smaller body weight gain in mice. The pyrosequencing showed that liraglutide, but not saxagliptin, substantially changed the overall structure of the gut microbiota as well as the relative abundance of weight-relevant phylotypes. Subsequent ridge regression analyses indicated that, in addition to food intake (β = −0.182, *p* = 0.043 in phylotypes inversely correlated with body weight) and blood glucose level (β = −0.240, *p* = 0.039 in phylotypes positively correlated with body weight), the administration of liraglutide was another independent factor associated with the abundance of weight-relevant phylotypes (β = 0.389, *p* = 6.24e-5 in inversely correlated ones; β = −0.508, *p* = 2.25e-5 in positively correlated ones). These results evidenced that GLP-1 receptor agonist liraglutide could modulate the composition of the gut microbiota, leading to a more lean-related profile that was consistent with its weight-losing effect.

Glucagon-like peptide-1 (GLP-1) is an incretin hormone which is secreted by L cells in the intestine in response to food ingestion^1^. This hormone can enhance glucose-induced insulin and suppress glucagon secretion. However, the natural intact GLP-1 is degraded rapidly, mainly via enzymatic inactivation by dipeptidyl peptidase-4 (DPP-4). Therefore, various GLP-1 receptor agonists (incretin mimetics) and DPP-4 inhibitors (incretin enhancers) have been developed for the clinical management of hyperglycaemia. Most recently, the GLP-1 receptor agonist liraglutide has been recognized as a promising anti-obesity agent for its ‘additional effect’ on weight loss in obese and/or diabetic individuals^2^,^3^. The DPP-4 inhibitors only mildly increase the concentration of active GLP-1 via inhibition of its degradation and do not result in significant body weight loss^4^.

Reduced food intake, resulting from inhibition of appetite and gastric emptying, has been suggested as the mechanism underlying the body weight control induced by GLP-1^1^,^5^. However, GLP-1 can induce more significant weight loss than that achieved by restricting the dietary intake alone. A randomized, double-blind, placebo-controlled study proved that, on the same 500 kcal per day energy-deficit diet and engaging in increased physical activity, the mean weight loss of participants receiving liraglutide was 2.1 kg to 4.4 kg greater than that of the patients receiving placebo^3^. This finding indicates that, besides reduced food intake, there are other mechanisms underlying the weight-loss effect of liraglutide that require further investigation.

Changes in the gut microbiota have been proven to be an important mediator of the development of obesity and related metabolic diseases as well as the secretion of incretin hormone. The obese microbiome is associated with an increased capacity to harvest energy from the diet, and this capacity is transmissible by the colonization of germ-free mice with the microbiota from obese individuals^6^. Germ-free mice transplanted with an endotoxin-producing strain isolated from the gut of an obese human developed obesity and insulin resistance on a high-fat diet^7^. Specific metabolites of gut microbiota may trigger the secretion of GLP-1 via the activation of G-protein-coupled receptors (GPRs)^8^. However, it remains to be elucidated whether and how the gut microbiota respond to GLP-1 and to what extend the shift correlates with its weight-control effect.

In the present study, we focused on the structural modulation of gut microbiota under enhanced incretin action. We performed pyrosequencing of 16s rRNA genes to generate comprehensive microbial community profiles under GLP-1 receptor agonist and DPP-4 inhibitor administration and further attempted to identify the key bacterial phylotypes that potentially play roles in the weight controlling effects of GLP-1.

## Results

### Blood glucose, body weight and metabolic profiles

**Figure 1:**
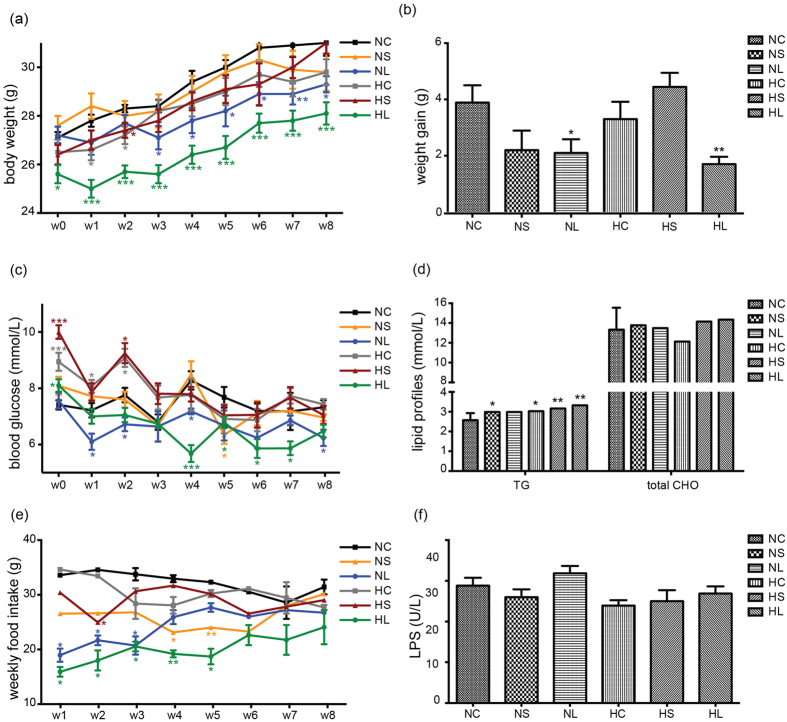
Characteristics of the mice in the six groups. The body weight (**a**) weight gain (**b**) blood glucose (**c**) lipid profile (**d**), food intake (**e**) and LPS concentration (**f**) of the mice in the six groups. NC, normal glucose control, n = 9; NS, normal glucose + diet premixed with saxagliptin, n = 10; NL, normal glucose + daily subcutaneous injection of liraglutide, n = 10; HC, transient hyperglycaemia control, n = 10; HS, transient hyperglycaemia + diet premixed with saxagliptin, n = 9; HL, transient hyperglycaemia + daily subcutaneous injection of liraglutide, n = 9. The data were the means  ±  SE. *p < 0.05, **p < 0.01, ***p < 0.001 *vs.* NC group by an unpaired Student’s t-test.

Compared with the NC group, the mean body weight was significantly lower in the liraglutide-treated groups (NL *vs.* NC, 28.00 ± 0.37 g *vs.* 29.19 ± 0.25 g, *p* = 0.017; HL *vs.* HC, 26.60 ± 0.33 g *vs.* 28.32 ± ±0.43 g, *p* = 0.005, unpaired Student’s t-test) (Fig. 1a, Supplementary Table S1). Liraglutide significantly reduced the body weight gain at the end of the experiment in both normoglycaemic (NL *vs.* NC, 2.10 ± 0.48 g *vs.* 3.89 ± 0.61 g, *p* = 0.033, unpaired Student’s t-test) and hyperglycaemic mice (HL *vs.* HC, 1.70 ± 0.26 g *vs.* 3.30 ± 0.62 g, *p* = 0.034, unpaired Student’s t-test) (Fig. 1b, Supplementary Table S1). The random blood glucose level was significantly higher in HC mice than in NC group from week 0 to week 2 (week 0, 8.94 ± 0.32 mmol/L *vs.* 7.41 ± 0.17 mmol/L, *p* = 4.95e-4; week 1, 8.07 ± 0.22 mmol/L *vs.* 7.22 ± 0.26 mmol/L, *p* = 0.038; week 2, 9.08 ± 0.32 mmol/L *vs.* 7.76 ± 0.26 mmol/L, *p* = 0.020; unpaired Student’s t-test); it then declined gradually and was comparable to that in NC mice from the end of week 3 (Fig. 1c, Supplementary Table S1). The mean blood glucose level was significantly lower in liraglutide-treated mice compared with the control mice, who were fed *ad libitum* (6.70 ± 0.43 mmol/L *vs.* 7.62 ± 0.68 mmol/L, *p* = 9.0e-6, unpaired Student’s t-test), and no hypoglycaemia was observed (Supplementary Table S1).

The mean food intake of the mice in the NS (26.07 ± 0.75 g *vs.* 32.42 ± 0.57 g, *p* = 1.0e-7, unpaired Student’s t-test), NL (24.36 ± 0.85 g *vs.* 32.42 ± 0.57 g, *p* = 1.0e-7, unpaired Student’s t-test), HC (30.38  ±  0.73 g *vs.* 32.42 ± 0.57 g, *p* = 0.036, unpaired Student’s t-test), HS (28.14 ± 1.04 g *vs.* 32.42 ± 0.57 g, *p* = 0.001, unpaired Student’s t-test) and HL (20.11 ± 0.81 g *vs.* 32.42 ± 0.57 g, *p* = 1.0e-7, unpaired Student’s t-test) groups were all significantly lower than that of the mice in the NC group, while the mice being treated with liraglutide ate the least (Fig. 1e, Supplementary Table S1). There were no substantial differences in the total cholesterol and LPS concentrations among the six groups, while the triglyceride level of the transiently hyperglycaemic mice was considerably higher than that in the NC group (Fig. 1d,f, Supplementary Table S1).

### Characteristics of the pyrosequencing results

A total of 370,482 high-quality sequences and 974 OTUs (97% similarity) were obtained from the 57 samples through a 454 pyrosequencing analysis, with an average of 5,079 reads and 334 OTUs per sample (Table 1). These reads/OTUs were assigned to 10 different phyla, and the dominant bacterial phyla of all groups were Firmicutes, Bacteroides and Proteobacteria. The Ace and Chao estimators (Table 1), rarefaction curves and Shannon-Wiener curves indicated that there was similar richness and sufficient sequence coverage in all samples (Supplementary Fig. S2).

Further statistical analysis of the Shannon (*p* = 0.041, HC *vs.* NC, unpaired Student’s t-test) and Simpson (*p* = 0.030, HC *vs.* NC, unpaired Student’s t-test) indices showed that transient hyperglycaemia significantly reduced the overall microbial diversity (Table 1), even though the mild hyperglycaemia lasted for only three weeks in this study. Interestingly, the effect of GLP-1 augmentation on the microbial diversity seemed to be dependent on the glycaemic status of the mice. The bacterial diversity declined significantly in normoglycaemic mice treated with liraglutide (NL *vs.* NC, *p* = 4.70e-5 for the Shannon index, *p* = 2.44e-4 for the Simpson index, unpaired Student’s t-test) and saxagliptin (NS *vs.* NC, *p* = 0.009 for Shannon, *p* = 0.015 for Simpson, unpaired Student’s t-test) administration, whereas it rose to the normal level in transiently hyperglycaemic mice (Table 1).

The reduction in the microbial diversity observed in the NL group could be attributed to the more prominent enrichment of Firmicutes (69.08% in NL *vs*. 33.61% in NC, *p* = 0.001; 49.06% in HL *vs*. 33.61% in NC, *p* = 0.002; 69.08% in NL *vs*. 49.06% in HL, *p* = 0.001; metastats), and the parallel decrease in the proportion of Bacteroides (28.09% in NL *vs*. 59.89% in NC, *p* = 0.001; 43.00% in HL *vs*. 59.89% in NC, *p* = 0.001; 28.09% in NL *vs*. 43.00% in HL, *p* = 0.002; metastats). The abundance of Proteobacteria (2.07% in NL *vs*. 5.46% in NC, *p* = 0.011, metastats), Deferricateres (0.02% in NL *vs*. 0.16% in NC, *p* = 0.011, metastats) and Actinobacteria (0.04% in NL *vs*. 0.11% in NC, *p* = 0.011, metastats) in the NL group was also significantly decreased but was comparable to the NC in the HL group.

### The microbial structure of liraglutide-treated mice differed substantially from that of the other mice

**Figure 2:**
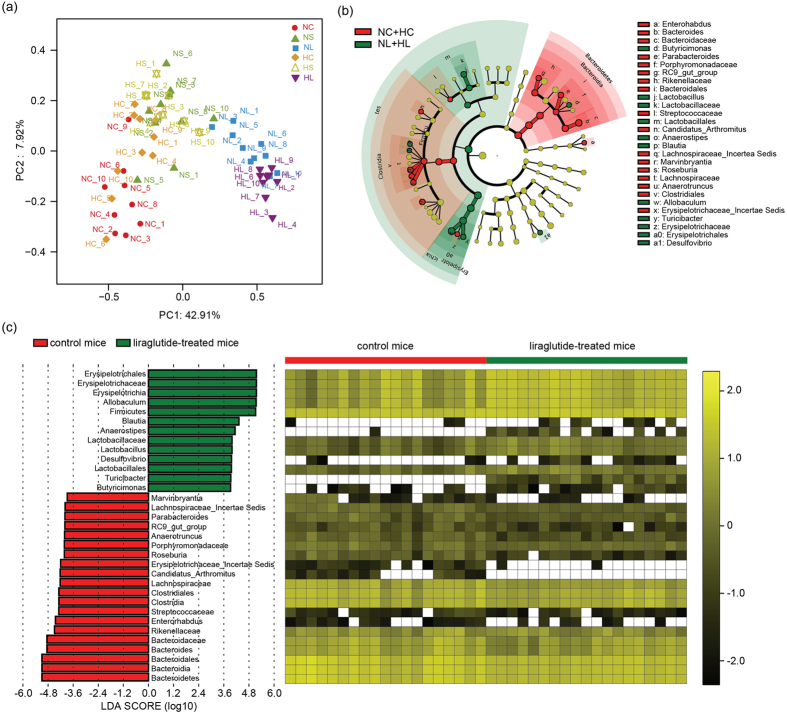
The overall structure and composition of the gut microbiota in liraglutide-treated mice differed significantly from that in the other group. PCoA plots based on unweighted Unifrac metrics (**a**) Each symbol represents a sample in from the NC, NS, NL, HC, HS and HL groups, respectively. The cladogram depicts the phylogenetic distribution of microbial lineages in faecal samples from mice with or without liraglutide treatment (**b**) Differences are represented in the colour of the most abundant group. Key phylotypes of the gut microbiota responding to liraglutide treatment (**c**) The left histogram shows the lineages with LDA values of 3.8 or higher as determined by LEfSe. The right heat map shows the relative abundance (log_10_ transformation) of OTUs.

To determine whether the liraglutide-mediated reduction of body weight is associated with alterations in the gut microbiota, we first profiled the overall microbial structure from mice in the six different groups. The results of PCoA based on an unweighted Unifrac distance matrix showed that there was a substantial rearrangement of the bacterial structure in liraglutide-treated mice compared to the control. Although no considerable glycaemia-related shift in the gut microbiota was observed, liraglutide showed a more prominent impact on the overall microbial architecture in the transiently hyperglycaemic mice relative to the separated microbiota clusters of normoglycaemic mice (Fig. 2a).

We further used a LEfSe analysis to discover the key variables that separated the gut microbiota under liraglutide treatment and identified 33 phylotypes as high-dimensional biomarkers. Thirteen of these phylotypes were increased, and 20 were decreased under liraglutide administration compared with the control group. The enriched phylotypes were the genera *Allobaculum (p* = 0.004, LEfSe) and *Turicibacter (p* = 1.77e-8, LEfSe) within the family *Erysipelotrichaceae*, the genera *Anaerostipes (p* = 5.51e-5, LEfSe) and *Blautia (p* = 0.039, LEfSe) within the family *Lachnospiraceae*, genus *Lactobacillus (p* = 0.013, LEfSe) within the family *Lactobacillaceae*, genus *Butyricimonas (p* = 0.005, LEfSe) within the family *Porphyromonadaceae*, and the genus *Desulfovibrio (p* = 0.008, LEfSe) (phylum Proteobacteria, class *Deltaproteobacteria*). The decreased phylotypes were mainly within the order *Clostridiales* (phylum Firmicutes) and the order *Bacteroidales* (phylum Bacteroides), for example, the genera *Candidatus_Arthromitus (p* = 7.17e-6, LEfSe), *Roseburia (p* = 5.63e-6, LEfSe) and *Marvinbryantia (p* = 6.38e-5, LEfSe) within the family *Lachnospiraceae* and the genus *Incertae Sedis (p* = 1.43e-6, LEfSe) within the class *Erysipelotrichia*. (Fig. 2b–d).

### The microbial composition in the transiently hyperglycaemic and saxagliptin-treated mice

Although the hyperglycaemia lasted for only three weeks, the microbial diversity of mice in the HC group was significantly reduced (*p* = 0.041 for the Shannon index; *p* = 0.030 for the Simpson index; unpaired Student’s t-test). The effect of saxagliptin on the microbial diversity exhibited the same tendency to decrease in normoglycaemic mice and was comparable to the normal level in the transiently hyperglycaemic mice (Table 1). However, no significant shift of the microbial composition was observed, as the clusters in four groups (NC, HC, NS and HS) could not be separated completely (Fig. 2a).

A taxonomy-based comparison was also performed. In saxagliptin-treated mice, the phylotypes mainly responsible for the increase of phylum Firmicutes were in the genus *Lactobacillus (p* = 0.023, LEfSe) within class *Lactobacillaceae* and also the genera *Allobaculum (p* = 0.017, LEfSe) and *Turicibacter (p* = 0.001, LEfSe) within class *Erysipelotrichaceae*. The decrease in the phylum Bacteroides was largely due to the genus *Bacteroides (p* = 0.003, LEfSe) within class *Bacteroidaceae* and the genus *Prevotella (p* = 0.018, LEfSe) within class *Prevotellaceae* (Supplementary Fig. S3).

There were no substantial changes in the relative abundance of the phyla Firmicutes and Bacteroidetes between the transiently hyperglycaemic mice and their normoglycaemic companions. The phylotypes responding to transient hyperglycaemia were mainly within the phylum Proteobacteria, for example, the genus *Helicobacter (p* = 0.001, LEfSe) within class *Betaproteobacteria* and the genus *Oscillibacter (p* = 0.041, LEfSe) within class *Epsilonproteobacteria*. Although not been identified as a high-dimensional biomarker, the abundance of the genus *Clostridium* also increased sharply (0.36% vs. 0.005%, *p* = 0.004, metastats) in the transiently hyperglycaemic groups. However, the relative abundance of these phylotypes was very low (<5%), indicating that the differences in the gut microbiota in normal mice and mice with transient hyperglycaemia were not substantial in the present study, which was consistent with the mixed clusters of these groups verified by the PCoA analysis (Fig. 2a, Supplementary Fig. S4).

### Identification of key OTUs associated with weight regulation

**Figure 3:**
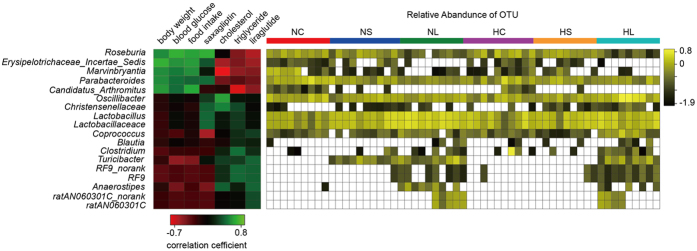
Correlation of the gut microbiota with body weight. The left heat map shows the correlation coefficients between the OTUs and various pathophysiological parameters. The right heat map shows the relative abundance (log_10_ transformed) of OTUs in the six groups.

The Spearman correlation of OTU counts (relative abundance) of bacterial families and genera with the body weight of mice was determined to identify key OTUs that were potentially relevant to weight in different groups. The phylotypes found to be significantly relevant to weight were affiliated with phyla Firmicutes (two families and 11 genera), Bacteroidetes (one family and two genera) and Tenericutes (one family and one genus). There were five genera associated with an increase in weight which were *Erysipelotrichaceae Incertae Sedis, Marvinbryantia, Roseburia, Candidatus Arthromitus*, and *Parabacteroides*. The phylotypes associated with a decrease in weight were the genera *Lactobacillus, Turicibacter, Anaerostipes, Coprococcus, Blautia, Oscillibacter* and *Clostridium*; the families *Lactobacillaceae, Christensenellaceae*, and *ratAN060301C*; and the only no-rank family within order *RF9* (Fig. 3, Table 2).

The relative abundance of all of the obesity-related phylotypes was substantially decreased under liraglutide administration, while only one of phylotypes (the genus *Candidatus Arthromitus*) was affected by saxagliptin. With respect to the lean-related phylotypes, both liraglutide and saxagliptin induced enrichment in the family *Lactobacillaceae* and the genera *Lactobacillus* and *Turicibacter*. In addition, liraglutide induced enrichment of the genus *Blautia*, and the genus *Coprococcus* was relatively enriched considering its decrease under saxagliptin administration (Figs 2 and 3, Table 2).

### Independent factors associated with the abundance of weight-relevant phylotypes

**Figure 4:**
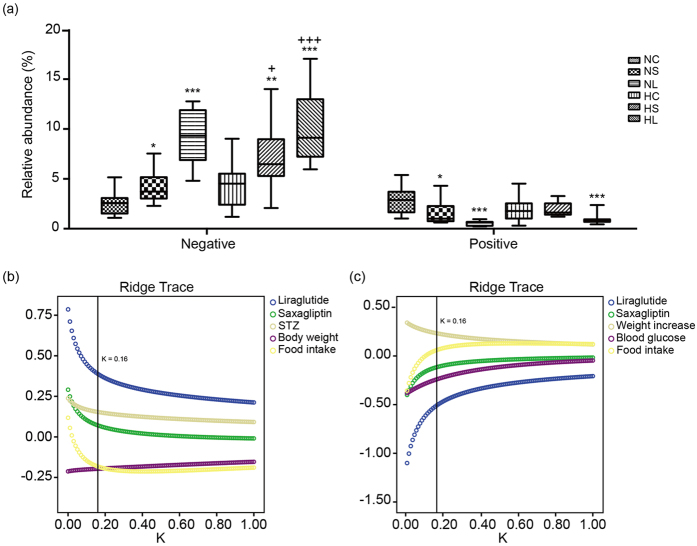
The abundance of and independent factors associated with weight-correlated phylotypes. The relative abundance of the phylotypes negatively and positively correlated with the body weight of mice in the six groups (**a**) The boundary of the box closest to zero indicates the 25th percentile, the line within the box marks the median, and the boundary of the box farthest from zero indicates the 75th percentile. Whiskers (error bars) above and below the boxes indicate the 90th and 10th percentiles. *p < 0.05, **p < 0.01, ***p < 0.001 *vs.* NC + *p* <0.05, +++ *p *< 0.001 *vs.* HC by the Mann-Whitney test. The standard ridge regression coefficient ridge trace of selected variables with an increase in the K for the abundance of phylotypes showed a negative correlation with body weight (**b**) and the abundance of phylotypes showed a positive correlation with body weight (**c**) The minimum K stabilizing all of the ridge traces of factors was 0.16.

Compared with saxagliptin, liraglutide showed a more prominent impact on the relative abundance of phylotypes correlated with body weight, regardless of whether they exhibited a negative or positive correlation (Fig. 4a). We then tried to identify the independent factors related to the relative abundance of weight-relevant phylotypes. Considering the abundances of weight-relevant phylotypes as dependent variables, and the administration of liraglutide, saxagliptin, STZ, body weight, increased body weight, the blood glucose level, food intake, and the concentrations of TG, CHO, and LPS as covariates, a ridge trace for each independent variable was drawn on a coordinate system. The variables with stable but small absolute values for the standard ridge regression coefficient or that had an unstable standard ridge regression coefficient verging infinitely to zero were removed^9^. The remaining variables were used to generate new ridge traces (Fig. 4b,c), and the contribution of the remaining variables was evaluated at the minimum K (0.16), which stabilized all of the ridge traces. The ridge regression analysis revealed that the abundance of lean-related phylotypes increased with liraglutide administration (β = 0.389, *p* = 6.24e-5) and reduced food intake (β = −0.182, *p* = 0.043), while the abundance of obesity-related phylotypes decreased with liraglutide administration (β = −0.508, *p* = 2.25e-5) and a higher blood glucose level (β = −0.240, *p* = 0.039).

## Discussion

Among the various efforts made to understand the mechanisms underlying the weight control effects of GLP-1, the current study is unique in that it focused on the changes in the gut microbiota induced by a GLP-1 receptor agonist and GLP-1 enhancer, and further determined whether these changes were associated with body weight loss.

We propose that moderate and transient hyperglycaemia is a condition in which extra weight loss resulting from severe hyperglycaemia is avoided. An analysis of the overall structures of the gut microbiota revealed no considerable glycaemia-related shifts associated with treatment, although specific phylotypes (mostly within the phylum Proteobacteria) were decreased. No substantial changes in the abundance of the phyla Firmicutes and Bacteroides were observed. This is different from previous reports indicating that the proportions of the phylum Firmicutes and the class *Clostridia* were considerably reduced in the guts of diabetic patients^10^. This discrepancy may be due to the mild level of hyperglycaemia in this study as well as the different model systems used (mice and humans).

Even with the similar reduction in food intake, saxagliptin had a neutral effect on body weight, while liraglutide induced significantly lower weight regardless of the glycaemic status, consistent with the results of previous studies^2^,^3^,^4^, although the impact of the stress induced by the daily injection of liraglutide could not be excluded. Previous studies reported that there was a correlation between the Firmicutes to Bacteroidetes ratio and obesity, with some of the studies indicating an increased ratio^11^,^12^, others indicating the inverse^13^, and recent studies have revealed no correlation at all^14^,^15^. The current study could not contribute to the debate because the Firmicutes to Bacteroidetes ratio increased under both liraglutide and saxagliptin administration, but only liraglutide induced a substantially lower weight gain, indicating that the family- and genus-level changes may be more relevant to body weight.

We then performed a correlation analysis to identify the phylotypes that were significantly correlated with body weight. The genera *Candidatus Arthromitus*^16^ (the yet to be cultured SFB^17^ *Parabacteroides*^18^, *Roseburia*^19^,^20^ and *Marvinbryantia*^21^ may contribute to weigh gain through their involvement in the host immunology^16^,^17^,^18^, improvement of fermentation and their specific products^19^,^20^,^21^. The genera *Lactobacillus*^22^,^23^ and *Coprococcus*^24^ are known to be associated with a low body mass index or high microbial richness in both animals and humans. However, not all of the weight-relevant phylotypes responded to the incretin augmentation. For example, the family *Christensenellaceae* is influenced by host genetics^25^, so its nonresponsiveness is conceivably due to the genetic background of the mice in the present study. There are also a few strains that seem to be incompatible with the body weight changes following liraglutide or saxagliptin exposure. For example, the abundance of the genus *Clostridium* (a lean-related phylotype) was significantly decreased under liraglutide treatment, especially in the transiently hyperglycaemic groups, indicating that this change in abundance resulted from both the blood glucose level and the incretin augmentation.

Although both liraglutide and saxagliptin target the incretin axis, liraglutide showed a more prominent impact on the abundance of weight-relevant phylotypes as well as the overall architecture of gut microbiota than saxagliptin. Compared with saxagliptin, liraglutide established a structurally rearranged architecture that may contribute to weight loss, with a profound impact on the weight-relevant phylotypes, especially on the obesity-related strains. Liraglutide decreased all of the obesity-related phylotypes and enriched five lean-related phylotypes, while also decreasing the genera *Roseburia, Erysipelotrichaceae Incertae Sedis, Marvinbryantia*, and *Parabacteroides* (obesity-related) while enriching genera *Blautia* and *Coprococcus* (lean-related). All of these changes were absent in the saxagliptin-treated mice, which could at least partly explain saxagliptin’s neutral role in body weight control.

The different effects of liraglutide and saxagliptin on gut microbiota may be due to their different extents of incretin augmentation. It has been shown that liraglutide (6 μg/kg, subcutaneous administration, once daily) increased the level of active GLP-1 to 60–90 pmol/L in humans, while single oral doses of sitagliptin (25 or 200 mg) increased the level of active GLP-1 to 10–20 pmol/L in rodents^26^,^27^. It is conceivable that liraglutide induced a four- to six-fold elevation in the active GLP-1 level relative to the DPP-4 inhibitor. Consistent with the differences in the active levels of GLP-1, the GLP-1 receptor agonist, but not the DPP-4 inhibitor, greatly delayed the gut transit time and gastric emptying rate^1^, and potentially affected the gut lumen internal environment, such as the local pH value and nutrient composition, which are factors known to affect the composition of the microbiota. Direct assays of the concentration of active GLP-1, the gastric emptying rate, and indices of the gut lumen internal environment should be performed in future studies, considering the fact that the effect of GLP-1 on the gut microbiota is likely ‘dose-dependent’ activity.

In the present study, we also explored the independent factors associated with weight-relevant phylotypes. Liraglutide, but not saxagliptin, was identified as an independent factor associated with both the phylotypes related to increased and decreased body weight. The other two independent factors were food intake (for the lean-related phylotypes) and the level of blood glucose (for the obesity-related phylotypes). Our results support the prior evidence that reduced food intake^28^ and hyperglycaemia^22^ modulate the structure of the gut microbiota. Previous studies attributed the weight-loss effects of GLP-1 receptor agonists to the reduction in food intake induced by the inhibition of appetite and gastric emptying^1^,^5^. Our present study provides the first evidence that a GLP-1 receptor agonist could modulate the composition of the gut microbiota, leading to a more lean-related profile that was consistent with the body weight changes. However, further investigation will be necessary to elucidate whether and to what extent the weight-loss effects of the GLP-1 receptor agonist are dependent on this modulation.

In the current study, we also examined the level of LPS, but no considerable difference was observed among the groups. Previous studies suggested the integral role of endotoxaemia-induced systematic, low-grade inflammation in the aetiology of obesity^22^,^29^. The comparable levels of LPS among the groups in our study indicated that the direct measurement of the plasma LPS level may not show any differences, but the concentration of LPS-binding protein (LBP) may be a more suitable marker. In fact, LBP may control the response to LPS by forming high-affinity complexes with LPS that bind to CD14 and trigger the downstream inflammation^28^,^30^.

This study demonstrated that the GLP-1 receptor agonist could modulate the gut microbiota to a more lean-related composition, indicating that GLP-1, as an incretin hormone, might be one of the limited factors (besides diet and oral drug intake, especially the intake of antibiotics) that can modulate the well-balanced host-microbial symbiotic status. As our current data were not generated in germ-free animal models and because faecal samples are not representative of the entire intestine, we anticipate that the modulation of the gut microbiota first documented here will be refined by more in-depth analyses, which may prove the ‘causative’ role of endogenous GLP-1 in regulating the gut microbiota and reveal the specific metabolic pathways in which the key microbiota are involved.

## Methods

### Animals and induction of transient hyperglycaemia

Sixty male ApoE -/- mice with a C57BL/6 genetic background (10 weeks old, Vital River Co. Beijing, China) were bred in a pathogen-free environment with a 12 h light/dark cycle and had free access to food and water. Animal experiments were performed in conformity with the National Institutes of Health Guide for the Care and Use of Laboratory Animals (NIH Pub. No. 85-23, revised in 1996) and were approved by the Animal Care and Use Committee of the Shanghai University of Traditional Chinese Medicine. When the mice were 11 weeks old, transient hyperglycaemia was induced by an intraperitoneal injection of streptozotocin (STZ) in 30 randomly selected mice. To avoid weight loss resulting from intense hyperglycaemia, a single dose of 60 mg/kg STZ was used. The blood glucose level was measured five days after the STZ injection to confirm that there was mild elevation.

### Experimental groups and metabolic profile

At the age of 12 weeks, mice with or without an STZ injection were further randomly divided into the following six groups: groups fed *ad libitum* that served as the controls (NC or HC), groups fed a diet premixed with saxagliptin (NS or HS), and groups that received a daily subcutaneous injection of liraglutide (NL or HL)(Fig. S1). Each group had 10 animals, and all groups were fed a high fat diet (Slaccas, Shanghai, China) containing 16.6% fat and 1.3% cholesterol for 8 weeks. The high-fat diet for the NS and HS groups was premixed with saxagliptin (Bristol-Myers Squibb, USA) to achieve a final concentration of 80 mg/kg (of the total dry diet, corresponding to 10 mg/kg of body weight). The NL and HL groups received a daily subcutaneous injection of liraglutide (Novo Nordisk, Denmark, 0.4 mg/kg/day) (Fig. S1). The body weight, random blood glucose level and food consumption of animals were monitored weekly. At the end of the eight-week period, the mice were isolated in individual metabolic cages and fasted for 16 hours, while faeces were collected and stored at −80 °C for further analysis. The plasma total cholesterol (CHO), triglyceride (TG) and lipopolysaccharide (LPS) concentrations were examined using a Bio-Swamp ELISA kit (Bio-Swamp, China) and were measured using the FlexStation 3^TM^ instrument (Molecular Devices, USA) according to the manufacturer’s instructions.

### DNA extraction, PCR and pyrosequencing

DNA was extracted from the mouse faeces using an E.Z.N.A. Stool DNA kit (Omega Bio-tek, Norcross, GA, USA) according to the manufacturer’s instructions. PCR amplification of the V1-V3 region of bacterial 16s rDNA was performed using universal primers (27F 5′-AGAGTTTGATCCTGGCTCAG-3′, 533R 5′-TTACCGCGGCTGCTGGCAC-3′) incorporating the FLX Titanium adaptors and a barcode sequence. The PCR amplification programme consisted of an initial denaturation step, followed by 25 cycles (annealing temperature at 55 °C), and a final extension step. Equal concentrations of amplicons were pooled from each sample. Emulsion PCR and sequencing were performed according to the recommendations of 454 Life Sciences, on a Roche Genome Sequencer GS FLX Titanium platform at Majorbio Bio-Pharm Technology Co., Ltd., Shanghai, China.

### Bioinformatics and multivariate statistics

A raw sequence was assigned to samples according to the barcodes, and the valid sequence was further trimmed according to the following rules: each pyrosequencing read contained no more than two mismatches in the forward primer and less than three mismatches in the reverse primer, each pyrosequencing read had no ambiguous reads and less than 10 homologous reads, chimeric sequences were excluded, and all sequences were more than 200 bp in length. Trimmed sequences were clustered to operational taxonomic units (OTUs) defined by 97% similarity using the UPARSE method^31^. The taxonomical assignment of OTUs was performed by alignment with the Bacterial SILVA database (SILVA version 115; http://www.arb-silva.de/documentation/release-115). OTUs that reached the 97% similarity level were used for diversity (Shannon, Simpson), richness (Chao, Ace), and rarefaction curve and Shannon-Wiener curve analyses using Mothur (version 1.30.1)^32^. Comparisons of diversity estimators and the metabolic indices between two groups were analysed by unpaired Student’s t-tests using SPSS 18.0. A heatmap figure was implemented by *R* packages gplots^33^ at the family and genus levels. An unweighted UniFrac distance metrics analysis was performed using the OTUs for each sample^34^. A principal coordinate analysis (PCoA) was conducted according to the matrix of distance. A Metastats analysis^35^ was used to detect significant changes in relative abundance of the microbial taxa between groups. The linear discriminant analysis effect size (LEfSe)^36^ was used to select OTUs that exhibited significance (LDA value = 3.8) in the structural segregation among the grouping of samples. Correlations between OTU counts and the body weight of mice were determined using Spearman correlations. Given that the GLP-1 receptor agonist inevitably reduced food intake^5^, a ridge regression analysis, which is thought to alleviate the effects of multicollinearity^9^, was performed to explore the independent variables for the abundance of weight-relevant phylotypes. The ridge regression was conducted based on a ridge regression function implemented in SPSS 18.0 (Ridge, Regression, Analyze). Q-value for false discovery rate (FDR) control^37^ was used for the multiple testing correction (by *R* packages qvalue^38^ for ridge regression).

### Data access

The 16s sequence data generated in this study were submitted to the NCBI Sequence Read Archive (SRA) database (accession number SRP057303).

## Additional Information

**How to cite this article**: Wang, L. *et al*. Structural modulation of the gut microbiota and the relationship with body weight: compared evaluation of liraglutide and saxagliptin treatment. *Sci. Rep.* **6**, 33251; doi: 10.1038/srep33251 (2016).

## Supplementary Material

Supplementary Information

## Figures and Tables

**Table 1:** A summary of the pyrosequencing data.

	NC (n = 9)	NS (n = 10)	NL (n = 10)	HC (n = 10)	HS (n = 9)	HL (n = 9)
Reads	4600 ± 159	5219 ± 277	5697 ± 161^***^	5189 ± 271	5137 ± 251	4537 ± 116
OTUs	339 ± 6	321 ± 15	317 ± 6^*^	320 ± 10	369 ± 12^*^	345 ± 17
Phylum	8	10	10	9	10	10
Class	14	16	16	15	16	16
Order	15	18	17	16	18	18
Family	28	31	32	31	35	34
Genus	47	50	51	50	54	53
Ace	437 ± 9	443 ± 19	458 ± 20	418 ± 12	477 ± 19	459 ± 14
Chao	437 ± 12	437 ± 20	431 ± 12	417 ± 12	467 ± 18	465 ± 16
Shannon	4.45 ± 0.08	3.87 ± 0.17^**^	3.70 ± 0.11^***^	4.06 ± 0.15^*^	4.43 ± 0.05	4.33 ± 0.06
Simpson	0.030 ± 0.004	0.089 ± 0.020^*^	0.102 ± 0.013^***^	0.064 ± 0.013^*^	0.037 ± 0.004	0.037 ± 0.003

Data are presented as the means ± SE. *p < 0.05, **p < 0.01, ***p < 0.001.

**Table 2:** The weight-relevant phylotypes were significantly different between the liraglutide- and saxagliptin-treated mice and their *ad libitum* companions.

		Spearman correlation		Liraglutide treated			Saxagliptin treated		
Phylotypes		*r* value	*p* value	Riched group	LDA (log10)	*p* value	Riched group	LDA (log10)	*p* value
phylum	Firmicutes								
class	Bacilli								
family	Lactobacillaceace	−0.320	0.015	NLHL	3.99	0.013	NSHS	3.98	0.023
genus	*Lactobacillus*	−0.320	0.015	NLHL	3.99	0.013	NSHS	3.98	0.023
class	Erysipelotrichia								
family	Erysipelotrichaceae								
genus	*Incerte Sedis*	0.428	0.001	NCHC	4.20	<0.001			
genus	*Turicibacter*	−0.314	0.017	NLHL	3.93	<0.001	NSHS	3.87	0.001
class	Clostridia								
family	Lachnospiraceae								
genus	*Marvinbryantia*	0.276	0.038	NCHC	3.88	<0.001			
genus	*Roseburia*	0.273	0.040	NCHC	4.02	<0.001			
genus	*Anaerostipes*	−0.286	0.031	NLHL	4.14	<0.001			
genus	*Coprococcus*	−0.322	0.015				NCHC	3.89	0.007
genus	*Blautia*	−0.292	0.028	NLHL	4.32	0.039			
family	Ruminococcaceae								
genus	*Oscillibacter*	−0.278	0.036						
family	Christensenellaceae	−0.278	0.036						
family	Clostridiaceae								
genus	*Candidatus_Arthromitus*	0.307	0.020	NCHC	4.22	<0.001	NCHC	4.08	< 0.001
genus	*Clostridium*	−0.412	0.001						
phylum	Bacteroidetes								
class	Bacteroidia								
family	Porphyromonadaceae								
genus	*Parabacteroides*	0.278	0.036	NCHC	3.99	0.003			
family	ratAN060301C	−0.334	0.011						
phylum	Tenericutes								
class	Mollicutes								
order	RF9	−0.439	0.001						
