# Supplementary material for: Structural modulation of the gut microbiota and the relationship with body weight: compared evaluation of liraglutide and saxagliptin treatment
Source: Sci Rep. 2016 Sep 16;6:33251. doi: 10.1038/srep33251 (PMC5025740; doi:10.1038/srep33251)
Supplement: Supplementary Information [file srep33251-s1.pdf]

Structural modulation of the gut microbiota and the relationship with body weight: compared evaluation of liraglutide and saxagliptin treatment

Lin Wang, Peicheng Li, Zhaosheng Tang, Xinfeng Yan, Bo Feng*

**Supplementary Information**

**Supplementary figure legends**

**Supplementary Figure S1.** The six groups: NC, normal glucose control; NS, normal glucose + diet premixed with saxagliptin; NL, normal glucose + daily subcutaneous injection of liraglutide; HC, hyperglycaemia control; HS, hyperglycaemia + diet premixed with saxagliptin; HL, hyperglycaemia + daily subcutaneous injection of liraglutide.

**Supplementary Figure S2.** The rarefaction curves (A) and Shannon-Wiener curves (B) of all of the samples.

**Supplementary Figure S3.** The cladogram (A) and the key phylotypes (B) of gut microbiota that changed in response to saxagliptin treatment. Differences are represented in the colour of the most abundant group. The left histogram shows the lineages of LDA values of 3.8 or higher as determined by LEfSe. The right heat map shows the relative abundance ($log_{10}$ transformation) of OTUs.

**Supplementary Figure S4.** The cladogram (A) and key phylotypes (B) of gut microbiota that changed in response to a different glucose level. Differences are represented in the colour of the most abundant group. The left histogram shows the lineages of LDA values of 3.8 or higher as determined by LEfSe. The right heat map shows the relative abundance ($log_{10}$ transformation) of OTUs.

**Supplementary Tables**

**Supplementary Table S1** Data on body weight, food intake, random blood glucose and blood lipid in
six groups.

| | Normal glucose | | | Transit hyperglycemia | | |
|---|---|---|---|---|---|---|
| | control (n=9) | Saxagliptin (n=10) | Liraglutide (n=10) | Control (n=10) | Saxagliptin (n=9) | Liraglutide (n=9) |
| Body weight (g) | | | | | | |
| Week 0 | 27.10±0.23 | 27.60 ± 0.40 | 27.20 ± 0.36 | 26.50±0.52 | 26.40±0.40 | 26.10±0.41$^{*}$ |
| Week 8 | 31.00±0.53 | 29.80 ± 0.77 | 29.30±0.34$^{*}$ | 29.80±0.53 | 31.00±0.44 | 28.10±0.46$^{**}$ |
| Mean weight | 29.19±0.25 | 29.00±0.58 | 28.00±0.37$^{*}$ | 28.32±0.43 | 28.68±0.34 | 26.60±0.33$^{**\#\#}$ |
| Weight gain | 3.89±0.61 | 2.20±0.70 | 2.10±0.48$^{*}$ | 3.30±0.62 | 4.44±0.50 | 1.70±0.26$^{*\#}$ |
| Foodintake (g/w) | | | | | | |
| Week 1 | 33.60±0.32 | 26.56±2.56 | 18.96±1.20$^{*}$ | 34.64±0.24 | 30.40±3.68 | 15.92±0.88$^{*}$ |
| Week 8 | 31.44±1.36 | 30.16±2.96 | 26.72±0.40 | 27.68±0.48 | 29.04±6.16 | 24.08±3.12 |
| Mean intake | 32.42±0.57 | 26.07±0.75$^{***}$ | 24.36±0.85$^{***}$ | 30.38±0.73$^{*}$ | 28.14±1.04$^{**}$ | 20.11±0.80$^{***}$ |
| Random blood glucose (mmol/L) | | | | | | |
| Week 0 | 7.41±0.17 | 8.09±0.32 | 7.56±0.28 | 8.94±0.32$^{***}$ | 10.00±0.24$^{***}$ | 8.10±0.22$^{*}$ |
| Week 1 | 7.22±0.26 | 7.72±0.12 | 6.10±0.29$^{*}$ | 8.07±0.22$^{*}$ | 7.87±0.31 | 7.00±0.26 |
| Week 2 | 7.76±0.26 | 7.60±0.28 | 6.72±0.24$^{*}$ | 9.08±0.32$^{*}$ | 9.25±0.36$^{*}$ | 7.05±0.25 |
| Week 3 | 6.80±0.27 | 6.68±0.56 | 6.64±0.54 | 7.67±0.43 | 7.81±0.37 | 6.74±0.15 |
| Week 8 | 7.33±0.25 | 6.96±0.13 | 6.24±0.29$^{*}$ | 7.43±0.19 | 7.05±0.32 | 6.48±0.22 |
| Mean level | 7.43±0.15 | 7.43±0.15 | 6.78±0.13$^{*}$ | 7.80±0.26 | 7.96±0.20$^{*}$ | 6.61±0.15$^{**\#\#}$ |
| Lipid (mmol/L) | | | | | | |
| TG | 2.58±0.13 | 2.97±0.10$^{*}$ | 2.99±0.15 | 3.04±0.09$^{*}$ | 3.16±0.12$^{**}$ | 3.32±0.11$^{**}$ |
| Total CHO | 13.34±0.78 | 13.77±0.67 | 13.49±0.83 | 12.14±0.70 | 14.15±0.72 | 14.34±0.53 |
| LPS (U/L) | 28.82±1.94 | 26.01±1.90 | 31.86±1.80 | 23.92±1.33 | 25.00±2.69 | 26.89±1.75 |

Data were presented as mean ± SE. *$p < 0.05$, **$p < 0.01$, ***$p < 0.001$ *vs.* NC. #$p < 0.05$, ##$p < 0.01$, *vs.* HC.

## Supplementary Figures

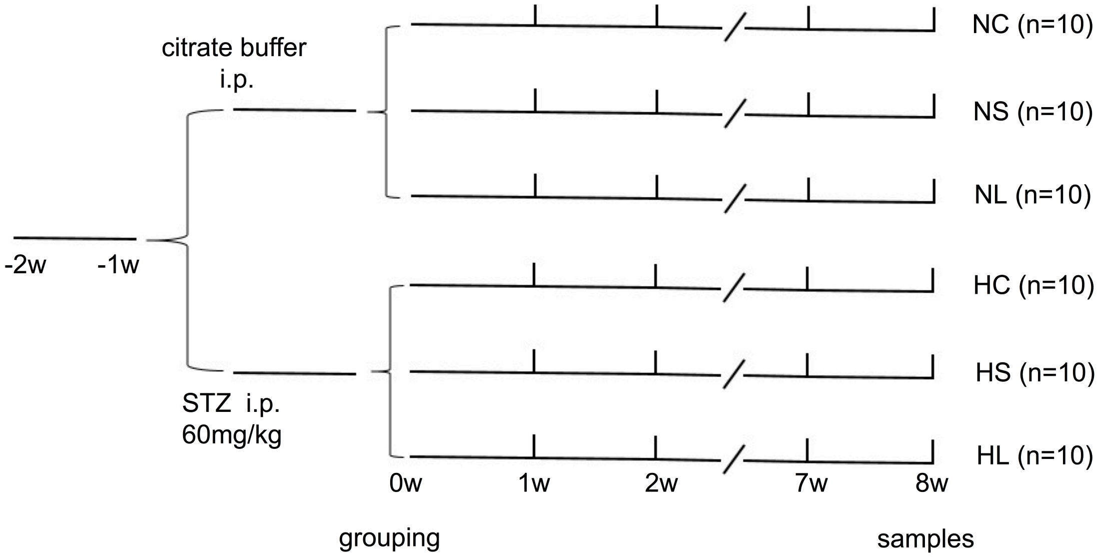


**Supplementary Figure S1.** The six groups.

(A)

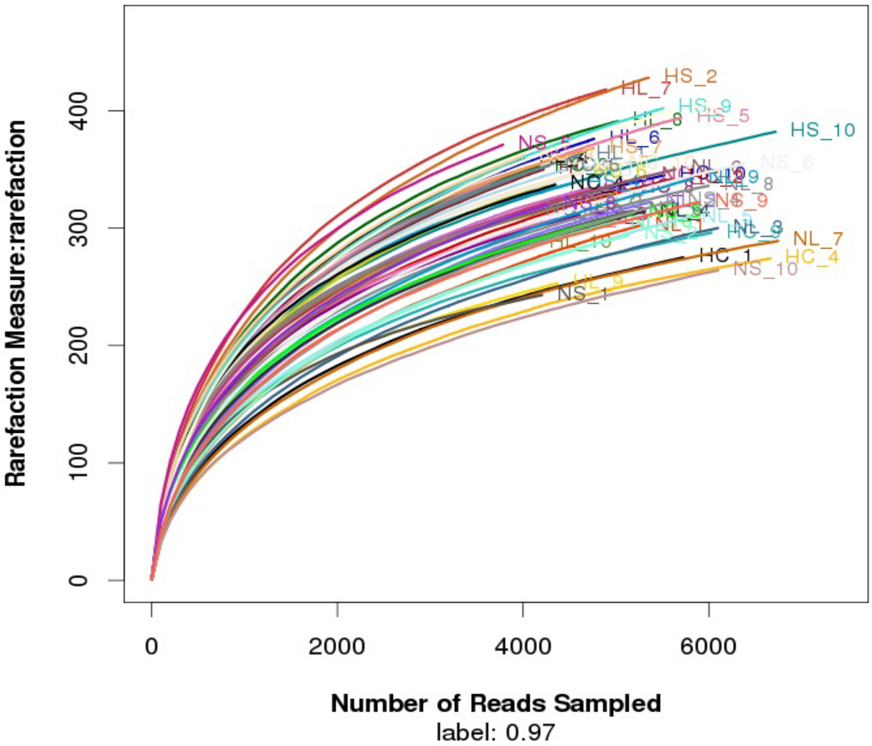


(B)

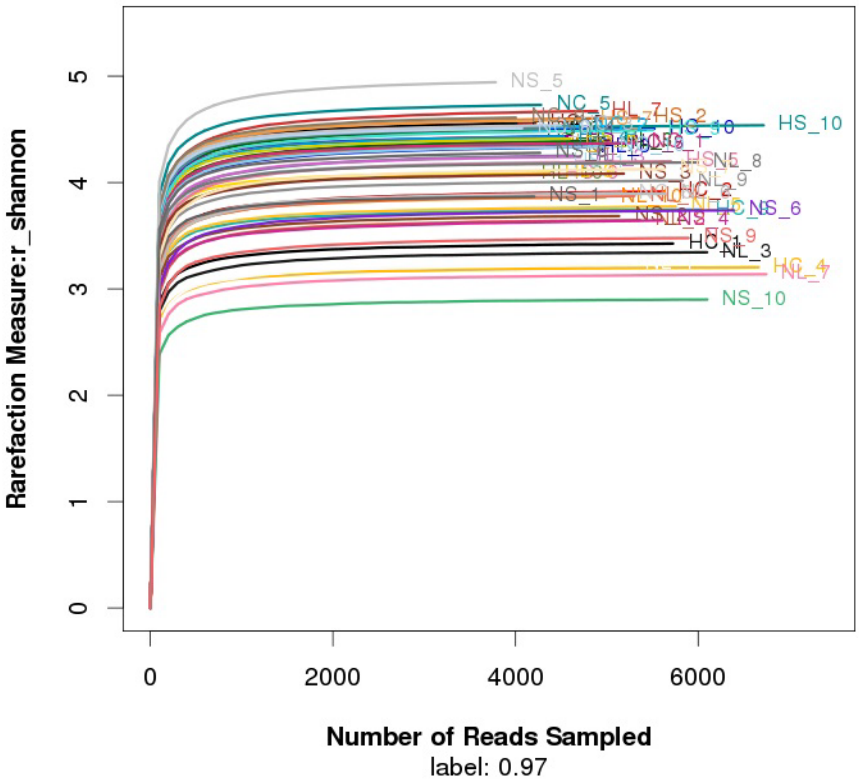


**Supplementary Figure S2.** The rarefaction curves (A) and Shannon-Wiener curves (B) of all of the samples.

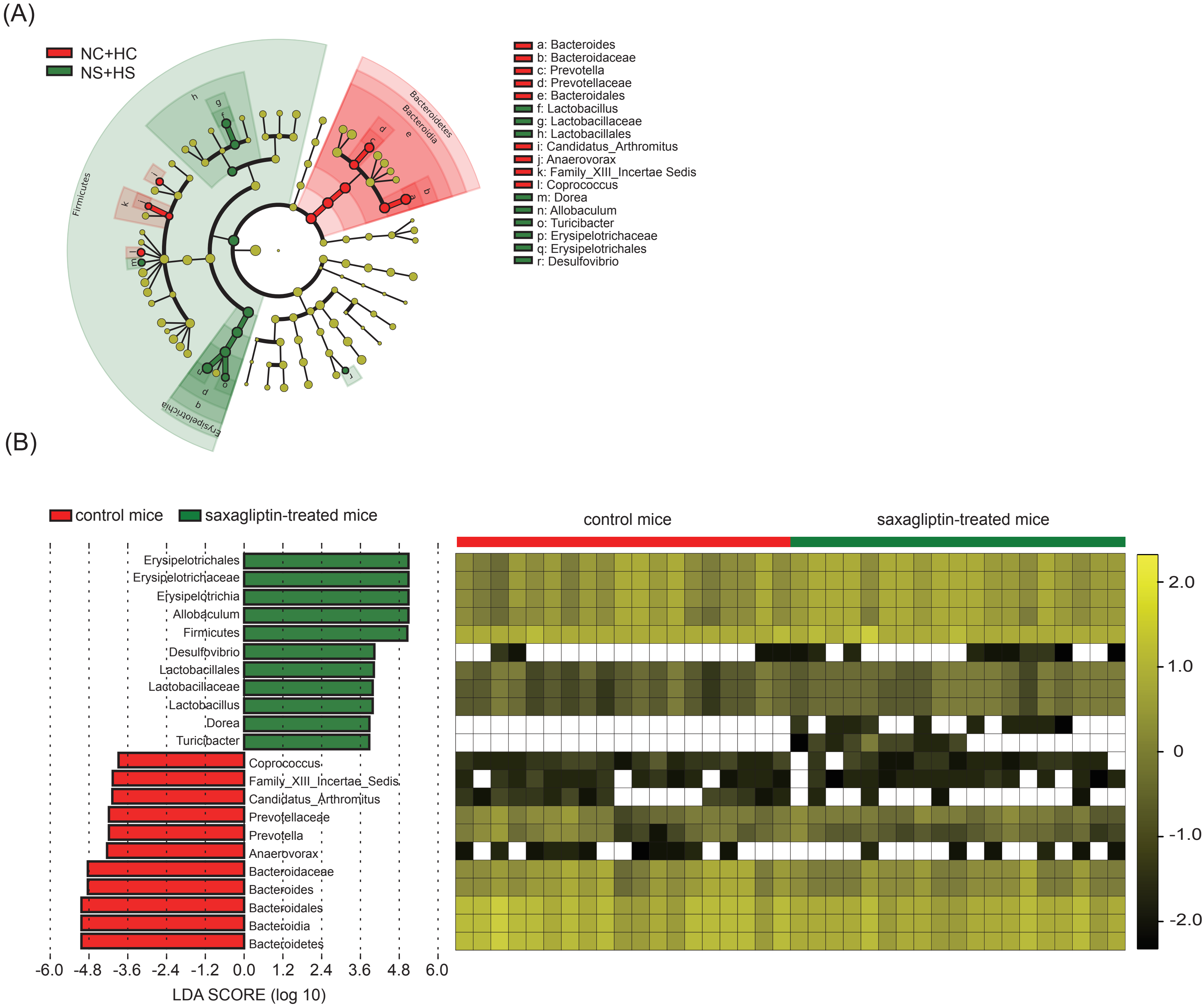


**Supplementary Figure S3.** The cladogram (A) and the key phylotypes (B) of gut microbiota that changed in response to saxagliptin treatment.

(A)

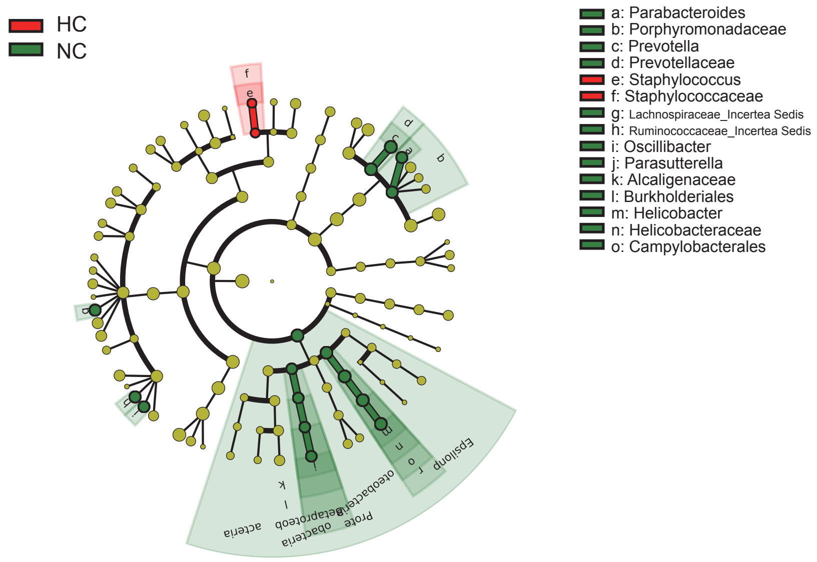


(B)

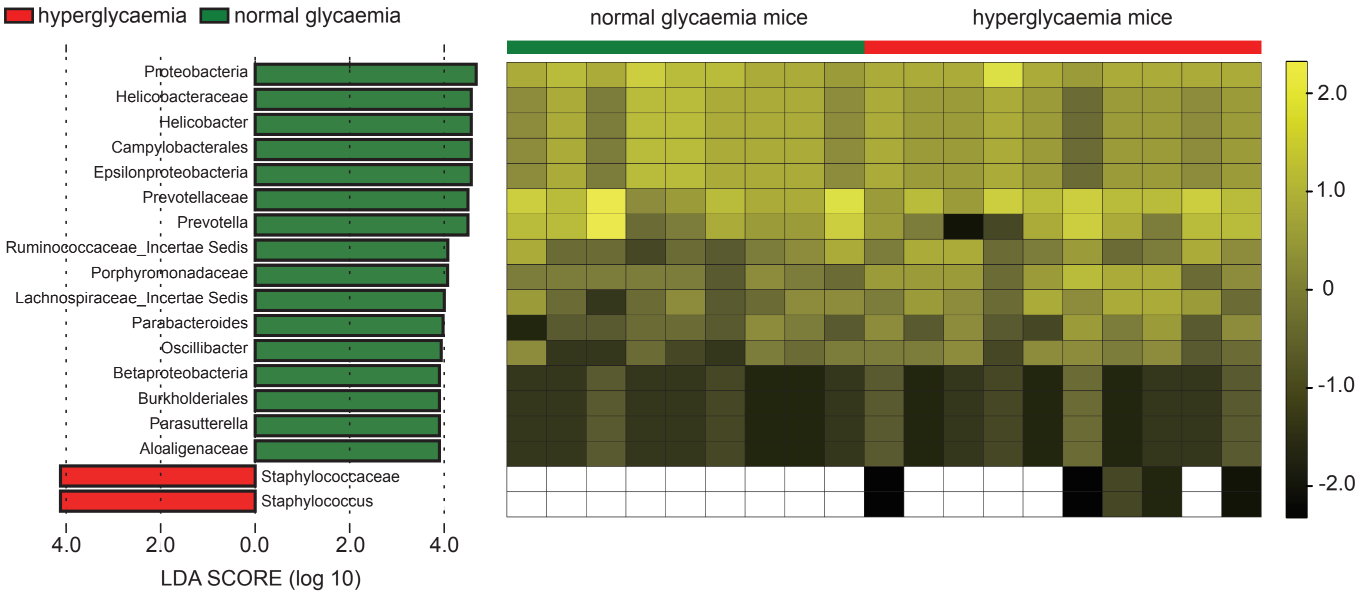


**Supplementary Figure S4.** The cladogram (A) and the key phylotypes (B) of gut microbiota that changed in response to a different glucose level.
